# A validation of the body compassion scale in females

**DOI:** 10.1177/13591053231160922

**Published:** 2023-03-16

**Authors:** Leesa M Van Niekerk, Gemma Muscella, Michael Quinn

**Affiliations:** University of Tasmania, Australia

**Keywords:** Body compassion, body image, reliability, self-compassion, validity

## Abstract

Body compassion bridges together the constructs of body image and self-compassion and refers to the relationship people have with their bodies, encompassing defusion, common humanity and acceptance. The purpose of the current study was to validate the 23-item Body Compassion Scale (BCS), in a female sample, and to determine the distinctiveness of body compassion from the similar construct of self-compassion. A total of 513 females completed an online cross-sectional survey examining body compassion, self-compassion, body image, and psychological health. Results supported the validation of the three-factor structure of the BCS, with the subscales of defusion and acceptance being more strongly associated with measures of body image than self-compassion. The BCS subscale of common humanity revealed lower associations with body image compared to self-compassion. The findings suggest that body compassion is a body-specific construct relating to body image concerns and while related, is distinct, from the construct of self-compassion.

## Introduction

Self-compassion is often incorporated within mind-body therapies and is viewed as a therapeutic target in and of itself, acting as a buffer to body image related concerns and psychological distress (Linardon et al., 2020). Self-compassion focuses on a general perception of how people view themselves in all aspects of life, comprises of the factors of self-kindness, common humanity, and mindfulness (Neff, 2003), and is not body-specific (Oliveira et al., 2018). To bridge the constructs of self-compassion and body image, with a view to providing a specific measure of body-related compassion, Altman et al. (2020) introduced the construct of body compassion. Body compassion comprises the three factors of defusion, common humanity and acceptance. Defusion involves removing evaluations about the bodies’ imperfections away from any notions of self-worth. Common humanity focuses on thoughts about the body that are collective within society, to reduce feelings of isolation. Finally, acceptance involves adopting a compassionate mindset about all aspects of the body (positive or negative) without judgement (Altman et al., 2020).

More recently, the role of body compassion has been explored in women with persistent health conditions and binge eating behaviours. In a cross-sectional survey, potential predictors of body compassion were examined in a sample of 227 women living with PCOS. The researchers found that higher levels of body compassion were associated with lower levels of body image concern as well as lower levels of depressive mood (Van Niekerk et al., 2022b). Van Niekerk et al. (2023) examined the level and predictors of body compassion in 318 women diagnosed with endometriosis and found that the level of distress associated with specific endometriosis-related symptoms was significantly associated with body compassion, indicating the potential utility of body compassion in combatting distress associated with physical health conditions. This demonstrates that body compassion may be a protective factor for those diagnosed with chronic health conditions (Van Niekerk et al., 2022a, 2023, under review), where encouraging women to feel more compassionate about their bodies may relate to psychological wellbeing. Barata-Santos et al. (2019) examined body compassion and the impact of major life events on binge eating behaviours in 458 Portuguese women and found that body compassion was negatively associated with both major life events and binge eating, with women higher in body compassion reporting less binge eating behaviours.

As body compassion is a relatively new construct, research investigating its distinctiveness from the similar construct of self-compassion is warranted. Whilst the construct of self-compassion also touches on notions of common humanity and acceptance, these themes are perceived more generally in all facets of life (Neff, 2003). In contrast, common humanity and acceptance within body compassion are conceptualised as being directly related to ideas about the body (Altman et al., 2020). Due to their conceptualisation, it is certainly expected that these two constructs will be somewhat associated, however it remains unclear whether body compassion is in fact distinct from self-compassion. Research investigating the claim that there is a distinct difference between self and body compassion is therefore fundamental to determine the distinctiveness of body compassion and to examine methods of assessment.

The Body Compassion Scale (BCS) was developed to provide a body-specific measure of compassion. The initial development of the BCS consisted of a 23-item scale to measure the three factors of defusion, common humanity and acceptance with good to excellent internal consistency as measured by Cronbach’s alpha and sound convergent validity across the three factors (Altman et al., 2020). To date, there has only been one further study that has explored the validation of the factor structure of the BCS. Policardo et al. (2022) explored the validity and reliability of an Italian language version of the BCS in a sample of 695 Italian women, with confirmatory factor analysis supporting the original three-factor structure of the BCS noted by Altman et al. (2020). Policardo et al. (2022) found the three subscales of the BCS to have very high internal consistency but that the covariance between acceptance and common humanity was weak (0.13), with no significant covariance between the defusion and common humanity (−0.07).

These findings differ from Altman et al. (2020) original study where all subscales revealed a significant moderate to strong covariance with each other. Additionally, Altman et al. (2020) initial development of the BCS investigated its utility in a limited participant sample of university students with a young mean age (20.24 years). The Policardo et al. (2022) study also utilised a young sample range (20–36 years). It is therefore important for research to further investigate the construct of body compassion, and its scale, in a broader population sample. Additionally, further research is needed to determine whether Altman et al. (2020) three-factor model, and its associated reliability and validity, holds across different samples, such as an Australian population sample.

### Aims and hypotheses

Although preliminary findings have demonstrated the applicability of body compassion across a range of clinical presentations (e.g. binge eating, persistent health conditions), research to determine the distinctiveness of body compassion from the construct of self-compassion, within a community sample of women, is still needed. Furthermore, additional research is warranted to validate the BCS (Altman et al., 2020) as a measure of body compassion. The current study therefore aims to investigate the factor structure of the BCS, complementing the literature on whether the three-factor model is the best fit for measuring the factors within body compassion. The current study also aims to contribute to the existing literature by broadening the sample characteristics of the test sample such as greater age range, educational and employment diversity. It is hypothesised that a three-factor model as described by Altman et al. (2020) will be supported. Body compassion, if distinct from the construct of self-compassion, will be theoretically more closely related than self-compassion to constructs measuring body-specific concepts (body image). It is therefore hypothesised that the correlations between body compassion and body image will be significantly higher than the correlations between self-compassion and body image measures.

## Methods

### Participants

Eligible participants were females aged 18 years and over with no current or prior persistent health condition or diagnosed eating disorder (self-reported). Responses were gathered from a total of 513 women (*M_age_* = 29.84 years, SD = 10.88) ranging from 18 to 68 years. The mean BMI for the sample, calculated from self-reported height and weight, was 25.73 (SD = 6.53), ranging from 15.61 to 47.02. This mean BMI is just above the healthy weight range for female adults (BMI 18.5–24.9) as determined by the Australian National Health and Medical Research Council (2013). Although Altman et al. (2020) included a small sample of male respondents in their original sample, research has cited significant differences in body image constructs across genders (Brennan et al., 2010) and, consistent with body image research that is not specifically measuring gender differences, a single gender group was chosen (female) for the current study. The female only sample was collected as part of a larger women’s persistent health research project that was completed by women only and is not indicative of body compassion having differing relevance across genders. Additional participant demographic information is displayed in Table 1.

**Table 1.** Participant demographic information.

Characteristic	*n*	%
Highest Academic Level Attained		
High school or below	26	5.1
College or equivalent (completed year 12)	145	28.3
Vocational certificate/qualification	69	13.5
Bachelor’s degree	169	32.9
Postgraduate degree	104	20.3
Current employment		
Student	205	40
Employed part-time	111	21.6
Employed full-time	147	28.7
Self-employed	22	4.3
Unemployed	20	3.9
Retired	8	1.6
Relationship Status		
Single	179	34.9
Casual	16	3.1
Committed, living separately	86	16.8
Committed, living together	96	18.7
Married, living together	136	26.5
Sexual Orientation		
Asexual	18	3.5
Demisexual	5	1
Bisexual	69	13.5
Heterosexual	398	77.6
Lesbian	17	3.3
Fluid	6	1.2
Menopause Status		
No symptoms of perimenopause	418	81.5
Have symptoms of perimenopause	52	10.1
Medical menopause	4	0.8
Surgical menopause	7	1.4
Natural menopause	32	6.2
Reproductive History		
Currently trying to conceive	17	3.3
Never been pregnant	327	63.7
Have been pregnant, no experience of miscarriage	101	19.7
Have been pregnant, 1 or more births and 1 or more miscarriages	61	11.9
Have been pregnant, each pregnancy has ended with miscarriage	7	1.4

*N* = 513.

### Procedure

This study was approved by the Tasmanian Social Sciences Human Research Ethics committee (REF: H0018163). Participants were recruited via social media advertisements as part of a larger study investigating compassion, health-related quality of life and psychological wellbeing. Additionally, an advertisement was placed on the University of Tasmania’s online recruitment system, SONA. Participants completed an anonymous cross-sectional survey online utilising the REDCap (Research Electronic Data Capture) platform (Harris et al., 2009). Participants provided demographic information including country of residence, sexual orientation, relationship status, educational attainment, and employment status. Information was also collected in relation to reproductive history, menopausal status, and height and weight. Survey logic required participants to select a response to each item prior to completion and submission. Participant consent to use their data was inferred upon submission of the questionnaire, as required by the HREC approval.

### Measures

The *Body Compassion Scale (BCS: Altman et al., 2020)* is a 23 item self-report measure assessing body compassion. The BCS measures three main factors of body compassion; defusion (e.g. ‘When my body is not responding the way I want it to, I tend to be tough on myself’), common humanity (e.g. ‘When I am frustrated with some aspects of my appearance, I try to remind myself most people feel this way at some time’) and acceptance (‘I am accepting of my looks just the way they are’). Items were reported on a 5-point Likert-type scale (1 = almost never, 5 = almost always). Items loading onto the defusion subscale are negatively worded, and were reverse scored (Altman et al., 2020). Higher scores on the BCS indicate higher levels of body compassion. The BCS has sound convergent validity with other measures (Policardo et al., 2022), and has also demonstrated excellent internal consistency in prior studies (Altman et al., 2020; Policardo et al., 2022).

The *Self-Compassion Scale (SCS: Neff, 2003)* is a 27 item self-report measure assessing self-compassion related to one’s life experience. Items are loaded onto one of six positive or negative domains including self-kindness (self-judgement), common humanity (isolation) and mindfulness (over-identification). An example item includes, ‘When I fail at something important to me, I become consumed by feelings of inadequacy’ (Neff, 2003). Items were rated on a 5-point Likert-type scale, based on level of agreement, (1 = almost never, 5 = almost always). A mean score is used to provide an overall score of self-compassion, where higher scores indicate higher self-compassion. The SCS considers scores to be low (1.0–2.49), moderate (2.5–3.5) or high (3.52–5.0) in self compassion (Neff, 2003). The SCS has demonstrated sound construct validity in both clinical and non-clinical samples (Costa et al., 2016; Raes et al., 2011). Cronbach’s α was 0.752 for the current sample.

The *Body Image Concern Inventory (BICI: Littleton et al., 2005)* is a 19-item self-report measure assessing dysmorphic body image concern. Items are rated on a 5-point Likert-type scale based on level of agreement (1 = not at all, 5 = completely), where higher scores indicate higher body image concern. An example item includes, ‘I am dissatisfied with some aspects of my appearance’ (Littleton et al., 2005). The clinical cut off score of the BICI is a total of 72, predictive of body dysmorphic concern. The BICI demonstrates good convergent validity with self-report measures investigating body image concerns, indicating the scale has sound predictive validity for anticipating body image concerns generally (Littleton et al., 2005). The BICI also demonstrates sound construct validity in an Australian university student sample (Collison and Mahlberg, 2019). Cronbach’s α was 0.951 for the current sample.

The *Body Attitude Test (BAT: Probst et al., 1995)* is a 20 item self-report measure assessing an individual’s attitude towards their own body. An example item includes, ‘I have a strong desire to be thinner’. Items are rated on a 6-point Likert-type scale based on level of agreement (0 = never, 5 = always). The BAT incorporates three subscales including, negative experience with body size, lack of familiarity with one’s own body and general body dissatisfaction (Probst et al., 1995). Higher scores indicate an increased negative attitude towards one’s body. The clinical cut off score for a nonclinical population is a total score of 36. Scores for nonclinical populations are: total score (M = 26.7, SD *=* 14.6), negative appreciation with body size (M = 8.6, SD *=* 6.6), lack of familiarity with one’s body (M *=* 85.7, SD *=* 4.4) and general body dissatisfaction (M = 6.8, SD* =*4.2; Probst, 1997). The BAT has previously demonstrated utility in samples of women with eating disorders but has also demonstrated utility within a community sample of women, therefore demonstrating sound construct validity (Brytek-Matera and Probst, 2014). Cronbach’s α was 0.898 for the current sample.

The *Patient-Reported Outcomes Measurement Information System (PROMIS-SF: Pilkonis et al., 2011)* measures were utilised to explore depression and anxiety related symptoms in adults (Pilkonis et al., 2011). The *PROMIS Emotional Distress Short Form* (PEDs) is an 8 item self-report measure assessing cognitive representations of depression in adults. Total scores range from 8 to 40, where higher scores indicate higher levels of depressed mood. The PEDS has strong convergent validity with other self-report measures of depressive symptom expression (Pilkonis et al., 2014). Cronbach’s α for the PEDs was 0.956 for the current sample. The *PROMIS Anxiety Short Form* (PAS) is a 7 item self-report measure assessing level of anxiety in adults. Scores ranged from 7 to 35 where higher scores indicated greater anxiety (Pilkonis et al., 2011). The PAS demonstrates good construct validity as well as sound convergent validity with other self-report measures of anxiety symptoms (Bushey et al., 2021). Cronbach’s α for the PAS was 0.952 for the current sample.

The *Physical Health Symptoms (PHQ-15: Kroenke et al., 2002)* is a measure assessing somatic symptom severity and concern in adults. The PHQ-15 explores a total of 15 different types of physical symptoms, presented in the past 7 days. Example items include ‘Over the last week, how often have you been bothered by menstrual cramps’ and ‘Over the last week, how often have you been bothered by headaches’, with respondents indicating ‘Not at All’, ‘Bothered a Little’, or ‘Bothered a Lot’. Scores range from 0 to 30, where higher scores indicate increased concern for presented symptoms (Kroenke et al., 2002). The PHQ-15 has good reliability and sound construct validity in nonclinical population samples (Kocalevent et al., 2013). Cronbach’s α was 0.816 for the current sample.

### Data Analysis

Data were analysed using SPSS-27 and Mplus 8.2 (Muthén and Muthén, 2017). Confirmatory Factor Analysis (CFA) was performed to provide confirmation of the three-factor model of the BCS. The sample size of 513 participants exceeds the suggested minimum sample size for CFA (*n* = 300) as outlined by Brown (2015) and Muthén and Muthén (2002), *n* = 150). Parameters of the CFA model were estimated using the robust weighted least squares (WLSMV) estimator. WLSMV was chosen due to the ordinal categorical nature of the Likert-type scales used in the BCS. Brown (2015) therefore recommends using WLSMV as the estimator with categorical variables, given its specific design for ordinal data. Additional fit indices were computed in Mplus to further assess goodness of fit for the model including, the Comparative Fit Index (CFI), Tucker-Lewis Index (TLI), and the Root Mean Square Error of Approximation (RMSEA). For both CFI and TLI, values of >0.95 are considered to indicate a good model-fit, whilst values >0.90 indicate an acceptable model fit (Brown, 2015). RMSEA values <0.06 indicate good fit, with 0.06 to 0.08 considered indicative of moderate fit, and 0.08 to 0.10 considered marginal fit (Brown, 2015). Internal consistency of the BCS subscales were analysed using McDonald’s omega calculated using the methods outlined by Hayes and Coutts (2020). Views vary regarding what values constitute an acceptable Omega value, though values >0.75 are usually considered to be acceptable (Watkins, 2017). Pearson correlation coefficients were calculated to explore the correlations between body compassion, self-compassion, and measures of body image. Correlation difference tests were examined to determine whether correlations between body compassion and measures of body image were significantly different to correlations between self-compassion and measures of body image. Additionally, partial correlations, controlling the variable of self-compassion, was examined to determine whether relationships between body compassion and measures of body image, were still present when controlling for the variance explained by self-compassion, therefore examining the distinctiveness of body compassion (Altman et al., 2020).

## Results

### Body compassion, self-compassion, and psychological health

Means and standard deviations for outcome measures of body compassion, self-compassion and psychological health are displayed in Table 2. The BCS total score for the sample was similar to the total score of 72.16 (SD = 15.83) in the Altman et al. (2020) study. The mean total score for self-compassion was within the moderate range of self-compassion (Neff, 2003). The mean standardised score for the PEDs depression score was just within the mild range of depressive symptoms. The mean standardised score for the PAS anxiety score was within the mild range of anxiety symptoms.

**Table 2.** Means and standard deviations of outcome measures of body compassion, self-compassion, body image and psychological health.

Measure	M (SD)	Sample range
Body compassion		
Defusion	28.57 (8.90)	9–45
Common humanity	25.93 (8.06)	9–45
Acceptance	14.81 (5.51)	5–25
Total score	69.32 (18.15)	23–114
Self-compassion		
Kindness	2.87 (0.85)	1–5
Humanity	3.07 (0.91)	1–5
Mindfulness	3.20 (0.84)	1–5
Judgement	2.82 (1.00)	1–5
Isolation	3.06 (1.00)	1–5
Over-identity	2.88 (1.00)	1–5
Total mean score	2.98 (0.71)	1.03–4.80
Body Image Concern Inventory		
Total Score	55.47 (17.44)	20–95
Body Attitude Test		
Negative appreciation	12.58 (8.71)	0–35
Lack of familiarity	11.21 (4.79)	0–30
General body dissatisfaction	10.37 (5.62)	0–20
Total Score	39.91 (18.15)	4–90
Psychological		
Depression^a^	55.22 (9.44)	37.10–81.10
Anxiety^b^	57.73 (9.83)	36.30–82.70
Somatic^c^	7.78 (4.94)	0–28

*N* = 513.

a PROMIS PEDs standardised scores.

b PROMIS PAS standardised scores.

c Somatic Concern PHQ-15 prorated total raw score.

### Body image

Means and standard deviations for the body image measures are also displayed in Table 2. The BICI total score for the present sample was below the clinical cut off score of 72, and similar to a total score of 50.4 (SD = 14.2) in a nonclinical university student sample in Littleton et al. (2005) study. The BAT total score for the present sample was below the average total score for Anorexia (M = 50.4, SD = 16.5) and Bulimia Nervosa (M = 69.4, SD *=* 22.0) populations, but slightly above the clinical cut off score of 36 for a nonclinical population (Probst, 1997). Although the score was slightly elevated it was well below the average scores noted in those with diagnosed eating disorders (Probst, 1997).

### Confirmatory factor analysis

**Figure 1.**
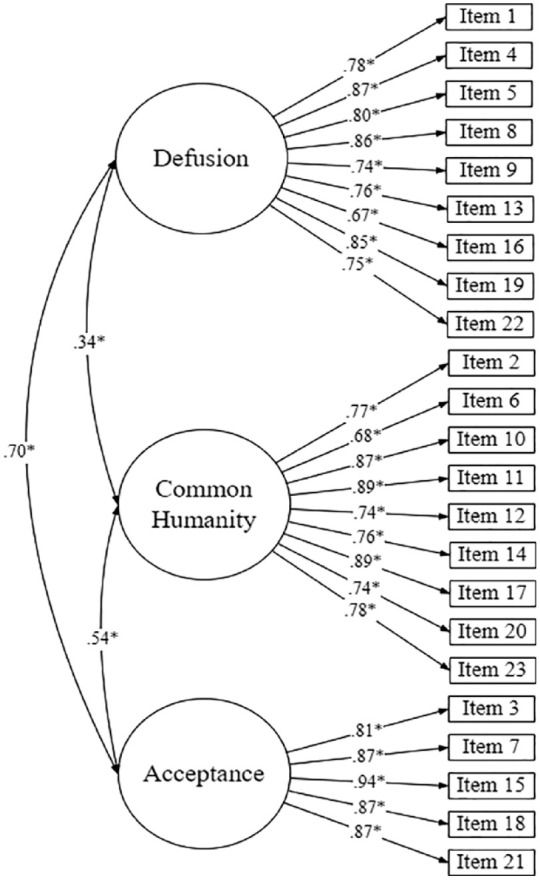
CFA model of the BCS, showing Standardised (STDYX) factor loadings and covariances between latent variables.

A CFA was conducted to validate the proposed three-factor model of the BCS. The 23 items were classified into the required corresponding factors *(defusion, common humanity*, and *acceptance)*, in accordance with Altman et al. (2020) three-factor structure. The chi-square for the model *χ*² (227) = 1141.856, p < 0.001, indicated model misfit. However, chi-square is affected substantially by sample size, and as such almost all models will be rejected if the *N* is large (Brown, 2015). Model fit indices were calculated with CFI and TLI showing acceptable to good fit, CFI = 0.951, TLI = 0.946, and RMSEA results demonstrating a marginal fit, RMSEA = 0.089, 90% CI [0.084, 0.094]. Standardised estimates for factor loadings and factor covariances are displayed in Figure 1. Standardised factor loadings ranged from 0.67 to 0.94, well above the minimum requirement of 0.50 to be considered meaningful (Costello and Osborne, 2005). Overall, these results findings suggest an acceptable to good fit for the three-factor structure of the BCS. There were substantive covariances between the latent factors, though they were low enough to suggest good discrimination. Overall, taken together, the CFA demonstrates that the three-factor structure of the BCS is valid and an acceptable to good fit.

### Internal consistency analysis of the BCS

Reliability was excellent for all subscales of the BCS as measured by McDonald’s omega (Defusion ω = 0.937; Common Humanity ω = 0.939; Acceptance ω = 0.942).

### Correlations

Bivariate correlation results revealed statistically significant weak to moderate correlations between body compassion, self-compassion, body image, somatic concern, depression, and anxiety (see Supplemental Table 1). Correlation difference tests explored whether there was a statistically significant difference between the correlations of body compassion with body image outcome measures, and those between self-compassion with body image outcome measures. The correlation between the *defusion* subscale and the BICI (*r* = −0.74) was significantly higher than the correlation between self-compassion and the BICI (*r* = −.59, *z* = 6.12, *p* < .001). Similarly, the correlation between the *acceptance* subscale and the BICI (*r* = −0.71) was significantly higher than the correlation between self-compassion and the BICI (*r* = −.59, *z* = −4.49, *p* < .001). However, the correlation between the *common humanity* subscale and the BICI (*r* = −0.29) was significantly lower than the correlation between self-compassion and the BICI (*r* = −.59, *z* = 7.89, *p* < .001). The correlation between *defusion* subscale and the BAT (*r* = −.71) was significantly higher than the correlation between self-compassion and the BAT (r = −.54, z = −6.47, *p* < .001). Similarly, the correlation between the *acceptance* subscale and the BAT (*r* = −0.72) was significantly higher than the correlation between self-compassion and the BAT, (r = −0.54, z = −6.68, *p* < .001). However, the correlation between the *common humanity* subscale and the BAT (*r* = −0.30) was significantly lower than the correlation between self-compassion and the BAT (*r* = −0.54, *z* = 6.15, *p* < .001). Taken together, the correlation difference tests show that body specific body compassion construct—at least the *defusion* and *acceptance* subscales thereof—is more closely related to body image than the self-compassion construct, as was hypothesised.

### Partial correlations

Partial correlations were examined to explore the correlations between the body compassion subscales of *defusion, common humanity* and *acceptance* and the body image outcome measures of the BICI and the BAT, controlling for the variance explained by self-compassion. Once variance explained by self-compassion was partialled out, the *defusion* subscale was still significantly negatively moderately correlated with the BICI, *r*_p_ (510510) = −0.572, *p* < 0.001 and the BAT, *r*_p_ (510) = −0.549, *p* < 0.001. Similarly, once variance explained by self-compassion was partialled out, the *acceptance* subscale was still significantly negatively and moderately correlated with the BICI, *r*_p_ (510) = −0.543, *p* = 0.001 and the BAT, *r*_p_ (510) = −0.586, *p* < 0.001. However, once variance explained by self-compassion was partialled out, the *common humanity* subscale was not significantly correlated with either the BICI, *r*_p_ (510) = 0.008, *p* = 0.858, or the BAT, *r*_p_ (510) = −0.039, *p* = 0.378.

Partial correlations controlling for BMI were also examined. The *defusion* subscale was still significantly negatively strongly with the BICI, *r*_p_ (510) = −0.741, *p* < 0.001, and the BAT, *r*_p_ (510) = −0.688, *p* < 0.001, when BMI was partialled out. The *common humanity* subscale was significantly negatively and weakly correlated with the BICI, *r*_p_ (510) = −0.278, *p* < 0.001 and the BAT, *r*_p_ (510) = −0.253, *p* < 0.001 when BMI was partialled out. The *acceptance* subscale was significantly negatively and strongly correlated with the BICI, *r*_p_ (510) = −0.72443, *p* < 0.001 and the BAT, *r*_p_ (510) = −0.695, *p* < 0.001, when BMI was partialled out.

## Discussion

The first aim of the study was to examine and validate the three-factor structure of the BCS in a community female sample. The three-factor structure of the BCS encompassing *defusion, common humanity* and *acceptance* was assessed by CFA, and showed acceptable to good fit. The present study also found good discrimination between the BCS latent factors demonstrating similarity in their relation to overall body compassion, providing further support for the three distinctive factor structure. The present study found a weak covariance between the *defusion* and *common humanity* factors, suggesting that the two constructs are not strongly associated with each other. The internal consistency of all BCS subscales was excellent as measured by both alpha and omega statistics. Taken as a whole, the present study therefore supports the overall factor structure, validity, and reliability of the BCS three-factor model.

With regards to convergent validity, the association between body compassion and measures of body image (BICI, BAT) suggests that overall, the BCS is theoretically related to measures of body image supporting the notion that body compassion is a body-specific construct (Altman et al., 2020). Similar to the findings of Policardo et al. (2022), the subscales of *defusion* and *acceptance* were negatively associated with body image measures, where higher levels of body compassion were associated with lower body image concerns. However, the subscale of *common humanity* was more weakly associated with measures of body image. Such results suggest that the factor of *common humanity* is perhaps not as ‘body-specific’ as originally theorised by Altman et al. (2020). Overall, partial support for the convergent validity of the BCS is suggested, given the weak associations between *common humanity* and measures of body image.

The second aim of the study was to examine the distinctiveness of body compassion in comparison to self-compassion. The results found that correlations between the body image measures and both the *defusion* and *acceptance* subscales of the BCS, were significantly higher than the correlations between measures of body image and self-compassion. This suggests that the subscales of *defusion* and *acceptance* are more body-specific than self-compassion, relating closer to measures of body image than self-compassion, as suggested by previous literature (Altman et al., 2020). This conclusion is supported by the partial correlations analysis which showed that the *defusion* and *acceptance* subscales had significant moderate to strong correlations with the BICI and the BAT, after controlling for self-compassion. This ultimately suggests that body compassion is a unique measure that is associated with measures of body image, over and above any variation explained by the self-compassion construct.

However, the *common humanity* subscale showed differing results. The correlation difference tests showed the correlations between *common humanity* and measures of body image were significantly *lower* than the correlations between self-compassion and measures of body image. Additionally, partial correlations controlling for self-compassion, found no significant association between *common humanity* with body image measures after controlling for self-compassion. This provides further support to the suggestion made above that *common humanity* may not be as ‘body specific’ in its current conceptualisation within the BCS. Indeed, the partial correlation results suggest that the *common humanity* subscale does not appear to measure much independent of self-compassion, in terms of its relationship with body image; indeed, the *common humanity* subscale may be more directly a measure of self-compassion, rather than body compassion. These results are also consistent across other analyses. Partial correlations controlling for BMI, showed that *defusion* and *acceptance* still had moderate to strong correlations with measures of body image. However, after partialling out BMI, *common humanity* was only weakly associated with measures of body image. These results were very similar to that of Policardo et al. (2022) study, showing that body compassion is still associated with measures of body image independent of BMI, but only for the *defusion* and *acceptance* subscales.

It can be presumed that these findings relating to the *common humanity* subscale are related to the development of the subscale itself. These items were originally derived from Neff’s (2003) Self-Compassion Scale (SCS), which also encompasses the elements of *common humanity. Common humanity* within self-compassion relates to the non-judgemental attitude surrounding perceived failures and inadequacies alongside the broader human experience (Neff, 2003). *Common humanity* items within the BCS were written to encompass the overarching themes of body-specific self-compassion to relate closer with body compassion. Despite this, although the items of *common humanity* are worded in a body-specific way, the items seem to continue to draw on the facets associated with *common humanity* within the construct of self-compassion, rather than within the construct of body compassion. Ultimately, the collective experience present within the factor of *common humanity* perhaps is not directly related to measures of body image (Policardo et al., 2022). Body image measures (BICI and BAT) have questions which focus on the individual perceptions and feelings about one’s own body. Both the BICI and the BAT refer to the individual experience of body image concerns or attitudes about the body and are not centred around a ‘shared experience’ with others. This contrasts with *common humanity* which is entirely related to the concept of a ‘shared experience’ with others. Taken together, this potentially explains the lack of association between *common humanity* and other measures of body image.

Given the findings pertaining to a lack of association between *common humanity* and body-specific measures, especially after controlling for self-compassion, further research should continue to explore the subscale of *common humanity* in its relation to body compassion overall. Potential reconceptualisation of *common humanity* or analyses exploring a two-factor model of *acceptance* and *defusion* may be useful in determining whether *common humanity* is a suitable construct that is body-specific within the broader body compassion construct. Nevertheless, the concept of *common humanity*, although relating closer with self-compassion, may still have utility in clinical samples. As *common humanity* focuses on reducing feelings of isolation, and acknowledging a shared experience (Altman et al., 2020), this concept may be an integral part to future treatment, where women with chronic health conditions may benefit from a shared experience of understanding with others. Body compassion was originally designed for intervention within health specific populations (Altman et al., 2020), where body compassion intervention may aid in the clinical prediction and treatment of body related concerns. In view of this, future research should continue to investigate the potential protective nature of body compassion and its clinical utility within perceived body image concerns in women. Ultimately, by exploring the utility of *common humanity* within the BCS, findings may suggest that *common humanity* may be beneficial factor when implementing intervention practices for specific clinical-health populations.

### Limitations and strengths

Notably, there are some limitations to the present study that should be considered. Research indicates that body image concerns are also prevalent amongst male populations (Griffiths et al., 2016). Male body image concerns surrounding physical appearance and muscularity (Griffiths et al., 2016) is an aspect which, generally, may not be as prevalent in body image concerns amongst women. Ultimately, one of the body image measures used in the present study (Body Attitude Test) was specifically designed for female populations. Examination of body compassion as a construct, and the utility of the BCS as a valid measure in male samples is therefore recommended for future research. Discriminant validity of the BCS was not explored as this study was part of a larger pre-existing study with outcome measures already included. Given the larger study had not been designed more broadly than just to validate the BCS, discriminant validity measures were not included. However, it is important to measure discriminant validity to ensure the BCS is not highly correlated with a measure it is theoretically not designed to be correlated with and further research is needed. The Impression Management subscale of the Balanced Inventory of Desirable Responding (Paulhus, 1994) has been used to measure discriminate validity is other body-related measures (Tylka and Wood-Barcalow, 2015). Further research is also required that includes a measure of positive body image to further delineate between self and body compassion. Although the present study had a broad age range of participants (18–68 years), the sample was still relatively young in relation to the mean age. Previous research exploring the validity of the BCS has also incorporated younger-aged populations (Policardo et al., 2022) therefore, it is difficult to ascertain how the construct of body compassion relates to older populations and further research in older populations is needed. Additionally, the survey was predominately advertised through various webpages on social media. This type of sampling will ultimately provide a biased sample of individuals who have access to the internet and are interested in webpages surrounding women’s health and body image.

## Conclusion

The documented association between body image concerns and decreased psychological wellbeing in women is apparent. Research exploring the validity and the utility of the body compassion construct is premature, yet initial research is promising in understanding the role of body compassion and its association with body image concerns in women. The current study extends on these findings by providing further validation of the BCS and evidence in support for the similarity, yet distinction, between self-compassion and body compassion as constructs, with further investigation on *common humanity* needed. Given the shift towards widely utilised mind-body therapies for targeting body image concerns, future research should continue to explore the protective nature of body compassion to enhance the clinical utility of this construct in targeting body-specific concerns in women.

## Supplemental Material

sj-docx-7-hpq-10.1177_13591053231160922 – Supplemental material for A validation of the body compassion scale in females Click here for additional data file. Supplemental material, sj-docx-7-hpq-10.1177_13591053231160922 for A validation of the body compassion scale in females by Leesa M Van Niekerk, Gemma Muscella and Michael Quinn in Journal of Health Psychology

sj-docx-8-hpq-10.1177_13591053231160922 – Supplemental material for A validation of the body compassion scale in females Click here for additional data file. Supplemental material, sj-docx-8-hpq-10.1177_13591053231160922 for A validation of the body compassion scale in females by Leesa M Van Niekerk, Gemma Muscella and Michael Quinn in Journal of Health Psychology

sj-out-6-hpq-10.1177_13591053231160922 – Supplemental material for A validation of the body compassion scale in females Click here for additional data file. sj-out-6-hpq-10.1177_13591053231160922 for A validation of the body compassion scale in females by Leesa M Van Niekerk, Gemma Muscella and Michael Quinn in Journal of Health Psychology This article is distributed under the terms of the Creative Commons Attribution 4.0 License ( http://www.creativecommons.org/licenses/by/4.0/) which permits any use, reproduction and distribution of the work without further permission provided the original work is attributed as specified on the SAGE and Open Access pages ( https://us.sagepub.com/en-us/nam/open-access-at-sage).

sj-sav-1-hpq-10.1177_13591053231160922 – Supplemental material for A validation of the body compassion scale in females Click here for additional data file. sj-sav-1-hpq-10.1177_13591053231160922 for A validation of the body compassion scale in females by Leesa M Van Niekerk, Gemma Muscella and Michael Quinn in Journal of Health Psychology This article is distributed under the terms of the Creative Commons Attribution 4.0 License ( http://www.creativecommons.org/licenses/by/4.0/) which permits any use, reproduction and distribution of the work without further permission provided the original work is attributed as specified on the SAGE and Open Access pages ( https://us.sagepub.com/en-us/nam/open-access-at-sage).

sj-sps-2-hpq-10.1177_13591053231160922 – Supplemental material for A validation of the body compassion scale in females Click here for additional data file. sj-sps-2-hpq-10.1177_13591053231160922 for A validation of the body compassion scale in females by Leesa M Van Niekerk, Gemma Muscella and Michael Quinn in Journal of Health Psychology This article is distributed under the terms of the Creative Commons Attribution 4.0 License ( http://www.creativecommons.org/licenses/by/4.0/) which permits any use, reproduction and distribution of the work without further permission provided the original work is attributed as specified on the SAGE and Open Access pages ( https://us.sagepub.com/en-us/nam/open-access-at-sage).

sj-sps-3-hpq-10.1177_13591053231160922 – Supplemental material for A validation of the body compassion scale in females Click here for additional data file. sj-sps-3-hpq-10.1177_13591053231160922 for A validation of the body compassion scale in females by Leesa M Van Niekerk, Gemma Muscella and Michael Quinn in Journal of Health Psychology This article is distributed under the terms of the Creative Commons Attribution 4.0 License ( http://www.creativecommons.org/licenses/by/4.0/) which permits any use, reproduction and distribution of the work without further permission provided the original work is attributed as specified on the SAGE and Open Access pages ( https://us.sagepub.com/en-us/nam/open-access-at-sage).

sj-sps-4-hpq-10.1177_13591053231160922 – Supplemental material for A validation of the body compassion scale in females Click here for additional data file. sj-sps-4-hpq-10.1177_13591053231160922 for A validation of the body compassion scale in females by Leesa M Van Niekerk, Gemma Muscella and Michael Quinn in Journal of Health Psychology This article is distributed under the terms of the Creative Commons Attribution 4.0 License ( http://www.creativecommons.org/licenses/by/4.0/) which permits any use, reproduction and distribution of the work without further permission provided the original work is attributed as specified on the SAGE and Open Access pages ( https://us.sagepub.com/en-us/nam/open-access-at-sage).

sj-spv-5-hpq-10.1177_13591053231160922 – Supplemental material for A validation of the body compassion scale in females Click here for additional data file. sj-spv-5-hpq-10.1177_13591053231160922 for A validation of the body compassion scale in females by Leesa M Van Niekerk, Gemma Muscella and Michael Quinn in Journal of Health Psychology This article is distributed under the terms of the Creative Commons Attribution 4.0 License ( http://www.creativecommons.org/licenses/by/4.0/) which permits any use, reproduction and distribution of the work without further permission provided the original work is attributed as specified on the SAGE and Open Access pages ( https://us.sagepub.com/en-us/nam/open-access-at-sage).

## References

[bibr1-13591053231160922] Altman JK Linfield K Salmon PG , et al. (2020) The body compassion scale: Development and initial validation. Journal of Health Psychology 25(4): 439–449. 28810491 10.1177/1359105317718924

[bibr2-13591053231160922] Australian National Health and Medical Research Council (2013) Clinical practice guidelines for the management of overweight and obesity in adults, adolescents and children in Australia. Available at: https://www.nhmrc.gov.au/about-us/publications/clinical-practice-guidelines-management-overweight-and-obesity#block-views-block-file-attachments-content-block-1

[bibr3-13591053231160922] Barata-Santos M Marta-Simões J Ferreira C (2019) Body compassion safeguards against the impact of major life events on binge eating. Appetite 134: 34–39. 30557589 10.1016/j.appet.2018.12.016

[bibr4-13591053231160922] Brennan MA Lalonde CE Bain JL (2010) Body image perceptions: Do gender differences exist? Psi Chi Journal of Psychological Research 15: 130–138.

[bibr5-13591053231160922] Brown TA (2015) Confirmatory Factor Analysis for Applied Research, 2nd edn. New York, NY: The Guilford Press.

[bibr6-13591053231160922] Brytek-Matera A Probst M (2014) Psychometric properties of the Polish version of the Body Attitude Test. Archives of Psychiatry and Psychotherapy 16: 39–46.

[bibr7-13591053231160922] Bushey MA Kroenke K Baye F , et al. (2021) Composite measures of pain, anxiety, and depressive (PAD) symptoms: Construct and predictive validity. General Hospital Psychiatry 72: 1–6. 34174547 10.1016/j.genhosppsych.2021.06.003

[bibr8-13591053231160922] Collison J Mahlberg J (2019) Psychometric evaluation of the body image concern inventory in an undergraduate sample. Clinical Psychologist 23: 112–123.

[bibr9-13591053231160922] Costa J Marôco J Pinto-Gouveia J , et al. (2016) Validation of the psychometric properties of the self-compassion scale. Testing the factorial validity and factorial invariance of the measure among borderline personality disorder, anxiety disorder, eating disorder and general populations. Clinical Psychology & Psychotherapy 23(5): 460–468. 26289027 10.1002/cpp.1974

[bibr10-13591053231160922] Costello AB Osborne J (2005) Best practices in exploratory factor analysis: Four recommendations for getting the most from your analysis. Practical Assessment, Research Evaluation 10(1): 1–9.

[bibr11-13591053231160922] Griffiths S Hay P Mitchison D , et al. (2016) Sex differences in the relationships between body dissatisfaction, quality of life and psychological distress. Australian and New Zealand Journal of Public Health 40(6): 518–522. 27372301 10.1111/1753-6405.12538

[bibr12-13591053231160922] Harris PA Taylor R Thielke R , et al. (2009) Research electronic data capture (REDCap)—a metadata-driven methodology and workflow process for providing translational research informatics support. Journal of Biomedical Informatics 42(2): 377–381. 18929686 10.1016/j.jbi.2008.08.010 PMC2700030

[bibr13-13591053231160922] Hayes AF Coutts JJ (2020) Use omega rather than Cronbach’s alpha for estimating reliability. but. . .. Communication Methods and Measures 14(1): 1–24.

[bibr14-13591053231160922] Kocalevent RD Hinz A Brähler E (2013) Standardization of a screening instrument (PHQ-15) for somatization syndromes in the general population. BMC Psychiatry 13: 91. 23514436 10.1186/1471-244X-13-91 PMC3606198

[bibr15-13591053231160922] Kroenke K Spitzer RL Williams JBW (2002) The PHQ-15: Validity of a new measure for evaluating the severity of somatic symptoms. Psychosomatic Medicine 64(2): 258–266. 11914441 10.1097/00006842-200203000-00008

[bibr16-13591053231160922] Linardon J Susanto L Tepper H , et al. (2020) Self-compassion as a moderator of the relationships between shape and weight overvaluation and eating disorder psychopathology, psychosocial impairment, and psychological distress. Body Image 33: 183–189. 32278251 10.1016/j.bodyim.2020.03.001

[bibr17-13591053231160922] Littleton HL Axsom D Pury CLS (2005) Development of the body image concern inventory. Behaviour Research and Therapy 43(2): 229–241. 15629752 10.1016/j.brat.2003.12.006

[bibr18-13591053231160922] Muthén LK Muthén BO (2002) How to use a Monte Carlo study to decide on sample size and determine power. Structural Equation Modelling 9(4): 599–620.

[bibr19-13591053231160922] Muthén LK Muthén BO (2017) Mplus User’s Guide, 8th edn. Los Angeles, CA: Muthén & Muthén.

[bibr20-13591053231160922] Neff KD (2003) The development and validation of a scale to measure self-compassion. Self and Identity 2(3): 223–250.

[bibr21-13591053231160922] Oliveira S Trindade IA Ferreira C (2018) The buffer effect of body compassion on the association between shame and body and eating difficulties. Appetite 125: 118–123. 29427690 10.1016/j.appet.2018.01.031

[bibr22-13591053231160922] Paulhus DL (1994). Balanced inventory of desirable responding: Reference manual for BIDR version 6. Unpublished manuscript, University of British Columbia, Vancouver, Canada.

[bibr23-13591053231160922] Pilkonis PA Choi SW Reise SP , et al. (2011) Item banks for measuring emotional distress from the patient-reported outcomes Measurement Information System (PROMIS®): Depression, anxiety, and anger. Assessment 18(3): 263–283. 21697139 10.1177/1073191111411667 PMC3153635

[bibr24-13591053231160922] Pilkonis PA Yu L Dodds NE , et al. (2014) Validation of the depression item bank from the patient-reported outcomes Measurement Information System (PROMIS®) in a three-month observational study. Journal of Psychiatric Research 56: 112–119. 24931848 10.1016/j.jpsychires.2014.05.010 PMC4096965

[bibr25-13591053231160922] Policardo GR Nerini A Di Gesto C , et al. (2022) Body Compassion Scale: A validation study in the Italian context. European Journal of Health Psychology 29: 88–98.

[bibr26-13591053231160922] Probst M (1997) Further experience with the body attitude. Test. Eating and Weight Disorders 2(2): 100–104. 14655849 10.1007/BF03339956

[bibr27-13591053231160922] Probst M Vandereycken W Coppenolle HV , et al. (1995) The body attitude test for patients with an eating disorder: Psychometric characteristics of a new questionnaire. Eating Disorders 3(2): 133–144.

[bibr28-13591053231160922] Raes F Pommier E Neff KD , et al. (2011) Construction and factorial validation of a short form of the self-Compassion Scale. Clinical Psychology & Psychotherapy 18(3): 250–255. 21584907 10.1002/cpp.702

[bibr29-13591053231160922] Tylka TL Wood-Barcalow NL (2015) The Body Appreciation Scale-2: Item refinement and psychometric evaluation. Body Image 12: 53–67. 25462882 10.1016/j.bodyim.2014.09.006

[bibr30-13591053231160922] Van Niekerk L Johnstone L Matthewson M (2022b) Predictors of self-compassion in endometriosis: The role of psychological health and endometriosis symptom burden. Human Reproduction 37(2): 264–273. 35102421 10.1093/humrep/deab257

[bibr31-13591053231160922] Van Niekerk LM Bromfield H Matthewson M (2022a) Physical and psychological correlates of self and body compassion in women with polycystic ovary syndrome. Journal of Health Psychology 27: 2566–2580. 34865539 10.1177/13591053211059390

[bibr32-13591053231160922] Van Niekerk LM Dell B Johnstone L , et al. (2023) Examining the associations between self and body compassion and health related quality of life in people diagnosed with endometriosis. Journal of Psychosomatic Research 167: 111202. 36812662 10.1016/j.jpsychores.2023.111202

[bibr33-13591053231160922] Watkins MW (2017) The reliability of multidimensional neuropsychological measures: From alpha to omega. Clinical Neuropsychologist 31(6-7): 1113–1126. 28429633 10.1080/13854046.2017.1317364

