# Supplementary material for: A validation of the body compassion scale in females
Source: J Health Psychol. 2023 Mar 16;28(10):900–12. doi: 10.1177/13591053231160922 (PMC10466952; doi:10.1177/13591053231160922)
Supplement: sj-docx-7-hpq-10.1177_13591053231160922 – Supplemental material for A validation of the body compassion scale in females [file sj-docx-7-hpq-10.1177_13591053231160922.docx]

**Explanatory Memo**

1. Data Files: *211205_Control_Full.sav* is the SPSS data file pertaining to the current manuscript.
2. SPSS Syntax: *BAT.sps, BICI.sps, BodyCompassion.sps* are the SPSS syntax files that were used to score the Body Attitude Test, the Body Image Concern Inventory, and the Body Compassion Scale.
3. Output: *OMEGA.out* is the output file for the factor analysis referred to in the submitted manuscript. *Preliminary_EFA_210820.spv* contains factor analysis output.
