# Supplementary material for: A validation of the body compassion scale in females
Source: J Health Psychol. 2023 Mar 16;28(10):900–12. doi: 10.1177/13591053231160922 (PMC10466952; doi:10.1177/13591053231160922)
Supplement: sj-docx-8-hpq-10.1177_13591053231160922 – Supplemental material for A validation of the body compassion scale in females [file sj-docx-8-hpq-10.1177_13591053231160922.docx]

**Supplementary Material**

**Supplementary Table 1**

*Pearson Correlations between Participant Characteristics and Outcome Measures of Body Compassion, Self-Compassion, Body Image and Psychological Health.*

|  | 1. | 2. | 3. | 4. | 5. | 6. | 7. | 8. | 9. | 10. | 11. | 12. |
| --- | --- | --- | --- | --- | --- | --- | --- | --- | --- | --- | --- | --- |
| 1. Age | - |  |  |  |  |  |  |  |  |  |  |  |
| 2. BMI | .181** | - |  |  |  |  |  |  |  |  |  |  |
| 3. PHQ-15 | -.275** | .149* | - |  |  |  |  |  |  |  |  |  |
| 4. PEDS | -.286** | .217** | .535** | - |  |  |  |  |  |  |  |  |
| 5. PAS | -.313** | .149* | .616** | .759** | - |  |  |  |  |  |  |  |
| 6. SC | .356** | -.162** | -.363** | -.605** | -.511** | - |  |  |  |  |  |  |
| 7. BC | .194** | -.302** | -.406** | -.516** | -.407** | .745** | - |  |  |  |  |  |
| 8. BC-DEF | .282** | -.213** | -.493** | -.589** | -.517** | .680** | .837** | - |  |  |  |  |
| 9. BC-HU | .069 | -.219** | -.151** | -.191** | -.108* | .500** | .745** | .315** | - |  |  |  |
| 10. BC-AC | .082 | -.329** | -.319** | -.467** | -.348** | .623** | .851** | .679** | .482** | - |  |  |
| 11. BICI | -.307** | .091* | .405** | .507** | .407** | -.590** | -.708** | -.740** | -.290** | -.711** | - |  |
| 12. BAT | -.156** | .271** | .393** | .517** | .405** | -.536** | -.696** | -.705** | -.297** | -.721** | .824** | - |

*Note. BMI= Body Mass Index; PHQ-15 = Somatic Health Symptoms; PEDS = PROMIS Emotional Distress Short-Form; PAS = PROMIS Anxiety Short Form; SC = Self-Compassion Scale; BC = Body Compassion Scale Total Score; BC-DEF= Body Compassion Defusion Subscale; BC-HU = Body Compassion Humanity Subscale; BC-AC = Body Compassion Acceptance Subscale; BICI = Body Image Concern Inventory; BAT = Body Attitudes Test; Significance level *p<.05 **p<.01.*
